# Mucosal-Associated Invariant T (MAIT) Cell Dysfunction and PD-1 Expression in Prostate Cancer: Implications for Immunotherapy

**DOI:** 10.3389/fimmu.2021.748741

**Published:** 2021-10-19

**Authors:** Ellie-May Jarvis, Shaun Collings, Astrid Authier-Hall, Nathaniel Dasyam, Brendan Luey, John Nacey, Gavin F. Painter, Brett Delahunt, Ian F. Hermans, Robert Weinkove

**Affiliations:** ^1^ Cancer Immunotherapy Programme, Malaghan Institute of Medical Research, Wellington, New Zealand; ^2^ Wellington Blood and Cancer Centre, Capital & Coast District Health Board, Wellington, New Zealand; ^3^ Department of Pathology and Molecular Medicine, University of Otago Wellington, Wellington, New Zealand; ^4^ Department of Surgery and Anaesthesia, University of Otago Wellington, Wellington, New Zealand; ^5^ The Ferrier Research Institute, Victoria University of Wellington, Wellington, New Zealand; ^6^ Immuno-oncology Programme, Maurice Wilkins Centre, Auckland, New Zealand

**Keywords:** prostate cancer, mucosal-associated invariant T cells, MR1 protein, PD-1 protein, human, pembrolizumab, natural killer T-cells, gamma delta T cells

## Abstract

Prostate cancer is the second most common cancer in men worldwide. Despite an abundance of prostate-specific antigens, immunotherapies have yet to become a standard of care, potentially limited by T-cell dysfunction. Up to 10% of human circulating T-cells, and a significant fraction in the urogenital tract, are mucosal-associated invariant T (MAIT) cells. MAIT cells express stereotyped T-cell receptors that recognize riboflavin metabolites derived from microbes presented by MR-1. We evaluated the number, phenotype and function of circulating MAIT cells, alongside two other innate-like T (ILT) -cell subsets, in men with prostate cancer and age- and sex-matched controls. MAIT cells in men with prostate cancer circulated at similar frequencies to controls, but their cytokine production and proliferation was impaired. In contrast, the function of two other ILT-cell populations (natural killer T-cells and Vγ9Vδ2 T-cells) was not impaired. In both patients and controls, MAIT cells expressed high levels of the immune checkpoint molecule PD-1 at rest, while upregulation of PD-1 in response to the MR-1 ligand 5-amino-6D-ribitylaminouracil (5-A-RU) was greater in patients. 5-A-RU also induced upregulation of PD-L1 and -L2 RNA in primary mononuclear cells. We confirmed that circulating MAIT cell number and function were preserved before and during anti-PD1 therapy with pembrolizumab in a cohort of patients with melanoma. *In vitro*, 5-A-RU enhanced mononuclear cell cytotoxicity against the PD-L1 positive prostate cancer cell line PC3 in an MR-1-dependent manner. Addition of pembrolizumab enhanced this cytotoxicity, and was associated with increased MAIT cell expression of CD107a and IFN-γ. We conclude that prostate cancer is associated with MAIT-cell dysfunction, and that this might be overcome through the application of potent MR-1 ligands with PD-1 blockade. These findings may have implications for the development of cancer immunotherapies that exploit MAIT cells.

## Introduction

Globally, prostate cancer is the sixth leading cause of cancer death among men, accounting for 359,000 deaths annually, a figure that is predicted to double over the next 20 years (1). Prostate cancer cells express tissue-specific antigens, which are potential targets for immunotherapies, including tumor vaccines or T-cell redirection (2, 3). However, late-phase clinical trials aimed at vaccinating against prostate-specific antigens have disappointed, with only Sipuleucel-T (an autologous dendritic cell therapy directed against prostatic acid phosphatase) demonstrating a statistically significant survival benefit (4). A role for checkpoint blockade immunotherapy in the treatment of prostate cancer is yet to be established, with little or no benefit of anti-CTLA-4 and/or anti-PD-1 therapies (5–11), possibly due to relatively low mutational burden and limited T-cell infiltration (12).

Innate-like T (ILT) -cells employ stereotyped T-cell receptors (TCRs) to recognize non-peptide antigens presented by molecules other than classical MHC, and together constitute a significant fraction of circulating T-cells. ILT-cells exhibit potent cytokine production and cytotoxicity, and their lack of MHC restriction means they can be specifically stimulated by conserved ligands, irrespective of patient tissue type (13). An ILT-cell population designated mucosal-associated invariant T (MAIT) -cells is especially prevalent in humans, alone constituting up to 10% of circulating human T-cells (14). MAIT-cells specifically recognize riboflavin metabolites produced by microbes presented on the molecule MR-1.

MAIT-cells have been detected in the urogenital tract (15), and appear to be involved in immune responses to urinary pathogens (16, 17). Notably *E. coli*, a common cause of urinary tract infection (18), is well recognized to activate MAIT-cells (19) and MAIT-cell dysfunction has been associated with recurrent urinary tract infection (20). Furthermore, transcriptomic analysis indicates the presence of MAIT-cells within prostate tissue, and suggests that infiltration of MAIT-cells into prostatic adenocarcinoma is associated with a favorable prognosis (21).

Dysfunction of T-cells, and ILT-cells in particular, has been described in a number of malignancies, and might limit the efficacy of cancer immunotherapies. For example, a numeric and functional deficit of natural killer T (NKT) cells, a minor ILT-cell population in humans, has been described in patients with prostate cancer (22). However, another ILT-cell subset known aas Vδ2^+^ gamma-delta T-cells, which recognize phosphoantigens, are numerically preserved in peripheral blood and present in tumour tissue in men with prostate cancer (23). This research aimed to determine the number and function of MAIT-cells in men with prostate cancer, and to explore their potential use for immunotherapy of prostate tumors. We report that circulating MAIT-cell numbers are preserved in prostate cancer, but that they exhibit impaired cytokine production and proliferative capacity upon specific stimulation, associated with enhanced expression of PD-1. We find that a potent MAIT-cell agonist can elicit MR-1-dependent lysis of a prostate cancer cell line, and that this is enhanced by PD-1 blockade. Our findings have implications for the rational design of immunotherapies that exploit MAIT-cells.

## Materials and Methods

### Human Samples

Ethical approval to use peripheral blood mononuclear cells (PBMCs) from healthy volunteers was provided by Victoria University of Wellington Human Ethics Committee (Ref: 20957) and approval to use patient and healthy control PBMCs was obtained from the University of Otago Ethics Committee (Ref: H15/114). Departmental research governance approval to recruit patients from Wellington Blood and Cancer Centre was sought and provided by the Clinical Research Unit at Capital & Coast District Health Board. In consideration of Te Tiriti o Waitangi (The Treaty of Waitangi), consultation with local iwi (tribes) *via* the Research Advisory Group Māori was undertaken (Ref: RAG-M 2015/413).

Healthy volunteers were recruited from Victoria University of Wellington. Study participants with cancer were recruited from oncology and/or urology clinics in the Wellington region, and healthy controls were referred by participants with cancer. Participant eligibility was assessed through a written questionnaire. Potential participants were excluded from the study if they were unable to provide a blood sample, were diagnosed with HIV or were taking immunosuppressant medication (not including cancer patients receiving chemotherapy). Those eligible to donate provided written informed consent.

Peripheral venous blood was drawn into EDTA vacuum tubes (BD) and PBMCs isolated *via* density gradient centrifugation using Lymphoprep (Axis-Shield). All PBMC samples were cryopreserved at -180°C in liquid nitrogen prior to batch assay analysis.

### Flow Cytometry

PBMCs were stained with fluorescent labelled antibodies, including CD3-BV510 (OKT3), CD4-BUV395 (SK3), CD8-PerCP-Cy5.5 (SK1), CD14-APC-Cy7 (63D3), CD19-BV785 or APC-CY7 (H1B19), CD107a-BV421 (H4A3), CD137-BV421 (4B4-1), CD161-FITC (HP-3G10), CD223-PE (11C3C65), CD274-PE (29E.2A3), CD279-BV421 or BV711 or PE (EH12.2H7), TCR-Vα24Jα18-APC (6B11), TCR-Vα7.2-BV605 (3C10), TCR-Vγ9-PE (B3), TCR-Vδ2-BV711 (B6), Ki67-PE (B56). The stained cells were characterized on the LSR II flow cytometer (Becton-Dickinson) and analysis performed using FlowJo software (V.10.7.1, Becton Dickinson). Relative fluorescence intensity (RFI) was calculated from the mean fluorescence intensity (MFI) as:

$RFI\;=\;\left(MFI_{stimulated}–\;MFI_{unstimulated}\right)/MFI_{unstimulated}$

### ILT-Cell Functional Analyses

In all assays MAIT-cells were identified as TCR-Vα7.2^+^CD161^+^CD3^+^CD19^-^ cells, and NKT and Vγ9Vδ2 T-cells were identified as TCR-Vα24Jα18^+^ CD3^+^CD19^-^ and TCR-Vγ9^+^TCR-Vδ2^+^ CD3^+^CD19^-^ cells, respectively. MAIT-cells were stimulated with 5-Amino-6D-Ribitylaminouracil combined 1: 1 with methyl-glyoxal immediately prior to use at a final concentration of 1 µM (5-A-RU/MG, Ferrier Institute, Lower Hutt, New Zealand). Vγ9Vδ2T and NKTs were stimulated with 1 μM disodium pamidronate (Hospira Australia Pty Ltd) and 200 nM α-galactosylceramide (α-GalCer, Ferrier Institute, Lower Hutt, New Zealand), respectively. Before use 5-A-RU was reconstituted from lyophilized powder in DMSO, diluted in PBS and stored at -20°C. MG, disodium pamidronate and α-GalCer were all stored at 4°C prior to use.

PBMCs were cultured with the above ILT-cell agonists and cell culture supernatant aspirated after 72-hours prior to the addition of 50 IU/mL interleukin-2 (IL-2, Sigma-Aldrich) to supplement T-cell growth. Cell culture supernatant cytokine analysis was performed using a Human Th1/Th2 Cytokine Bead Array, as per the manufacturer’s instructions (Bio-Rad Laboratories, Inc.). After a total of 7-days in culture at 37°C and 5% CO_2_, ILT-cell proportions and surface marker expression was analyzed by flow cytometry. ILT-cell expansion was determined per participant as the fold change in frequency relative to media control for each individual. In parallel, 10^5^ PBMCs were cultured for 48 hours and detection of IFN-γ Enzyme Linked Immuno-Spots (ELISpots) was performed using the IFN-γ ELISpot kit (Mabtech, Sweden) as per manufacturer’s instructions.

### RNA Nanostring

PBMCs from 6 healthy donors were cultured with 5-A-RU/MG or media control for 72 hours to specifically activate MAIT, NKT and Vγ9Vδ2T cells. RNA was extracted from these PBMCs using the Qiagen RNA mini kit (Qiagen) and purified using gDNA removal columns (Bio-Strategy) as per manufacturer’s instructions. The presence of RNA in samples was confirmed using the Nanodrop spectrophotometer (ThermoFisher Scientific PBMC). RNA was then applied to the PanCancer Immune nCounter Assay (NanoString Technologies, Inc.) according to the manufacturer’s instructions. The resulting raw data was normalized using nSolver (v3.0, NanoString Technologies, Inc.) Data was then log2-transformed and trimmed to exclude counts greater than 1.5 mean absolute deviations from the median value using RStudio (RStudio, Inc.).

### Impedance-Based Cell Cytotoxicity Assay

The American Type Culture Collection (ATCC; Manassas, USA) metastatic grade IV prostate cancer cell line ‘PC3’was cultured in 16-well gold electrode bottomed plates (E-plate 16, ACEA Biosciences) for 12 hours prior to the addition of effector cells of at a ratio of 20 effectors to 1 target cell. Impedance throughout the culture was measured using the XCelligence Real-Time Cell Assay (RTCA) software (ACEA Biosciences).

### Statistical Analyses

Statistical analyses of data were performed using Prism™ 8.3.0 (GraphPad Software, La Jolla, CA). Two-tailed P values were calculated in all analyses and a P value of < 0.05 was considered significant.

## Results

### Participant Characteristics

We recruited 36 men with prostate cancer from local oncology and urology clinics. Median age was 70.5 years. Half of patients had active prostate cancer and elevated Prostate Specific Antigen (PSA). Almost half of patients had metastatic prostate cancer, a third with castrate-refractory disease. All patients had received treatment for their cancer, with two thirds ever having radiotherapy or androgen deprivation therapy (**Table 1**). We recruited 20 healthy male controls with a median age of 71 years. Age was well matched between groups, although nine patients and no controls were taking systemic corticosteroids at the time of blood sampling; no participants were taking other immunosuppressants (**Supplementary Table 1**).

**Table 1:** Characteristics of participants with prostate cancer.

Characteristic	n (%)
**PSA (ng/mL)**	-
Within normal limits (≤4)	17 (47.2)
Elevated (>4)	19 (52.8)
**Metastatic Disease**	**16 (44.4)**
**Castrate Refractory**	**6 (16.7)**
**Ever received Androgen Deprivation Therapy**	24 (66.7)*
GnRH agonist	19 (52.8)
AR antagonist	3 (8.3)
Abiraterone	6 (16.7)
**Current Systemic Corticosteroid use**	**(9 25)**
Prednisone	7 (19.4)
Dexamethasone	2 (5.6)
**Prior radiotherapy**	**23 (63.9)**
External Beam	16 (44.4)
Brachytherapy	7 (19.4)
**Ever received Chemotherapy**	**5 (13.9)**

IQR, Interquartile Range. PSA, Prostate Specific Antigen. GnRH, Gonadotropin Releasing Hormone.

AR, Androgen Receptor. *Values add to greater than total due some patients using multiple agents. Indented row titles and values represent a subset of the parent characteristic above, in bold.

### Impaired MAIT-Cell Function in Men With Prostate Cancer

**Figure 1:**
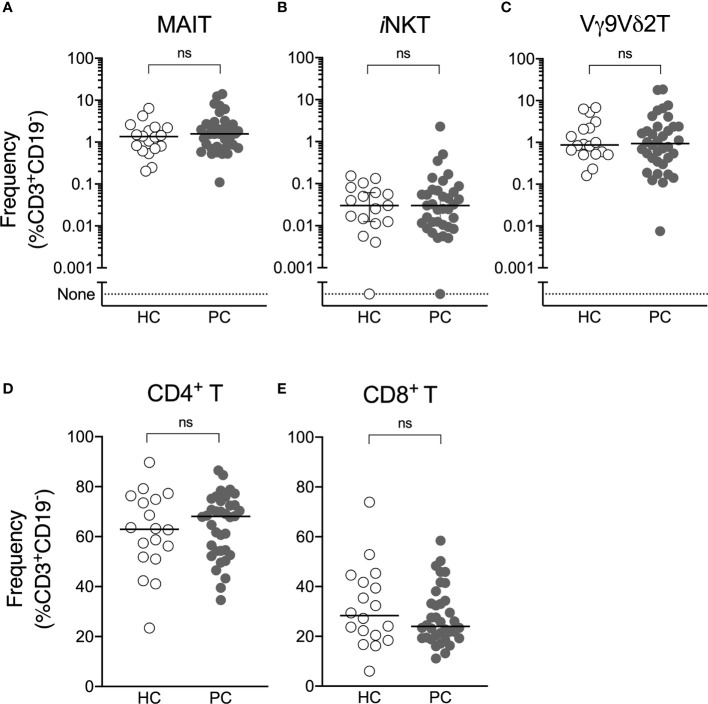
Circulating ILT-cell frequencies are preserved in men with prostate cancer. Flow cytometric analysis was used to determine the proportion of MAIT **(A)**, NKT **(B)**, Vγ9Vδ2T **(C)**, CD4^+^ **(D)** and CD8^+^ **(E)** T-cells between men with prostate cancer and healthy male controls. Mann-Whitney test. N = 20 healthy controls, 36 men with prostate cancer. ns, no significance.

The frequency of circulating ILT-cells was determined by flow cytometry (**Supplementary Figure 1**). We observed no difference in the circulating frequency of NKT, MAIT or Vγ9Vδ2 T-cells or conventional CD4^+^ and CD8^+^ T-cells between patients with prostate cancer and healthy controls (**Figure 1**).

**Figure 2:**
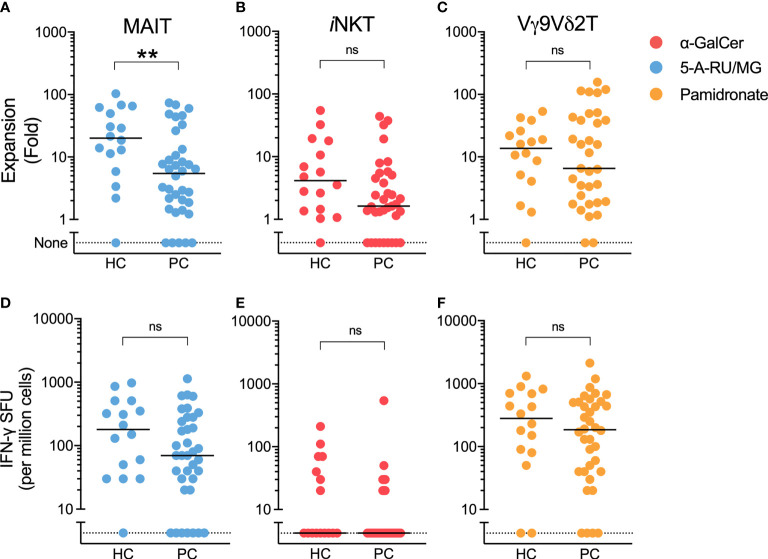
5-A-RU/MG stimulated MAIT-cell proliferation, but not IFN-γ SFU, is impaired in men with prostate cancer. The proliferation of MAIT **(A)**, NKT **(B)** and Vγ9Vδ2T **(C)** cells was measured at the end of 7 days in culture with stimuli as indicated and expressed as a fold change to media control. IFN-γ spot forming units (SFUs) determined by ELISpot assay are demonstrated for PBMCs cultured with 5-A-RU/MG **(D)**, α-GalCer **(E)** and pamidronate **(F)**. **P < 0.01, Mann-Whitney test. N = 20 healthy controls, 36 men with prostate cancer. ns, no significance.

Having found similar ILT-cell numbers in patients compared to controls, we next assessed ILT-cell function. Patient and control PBMCs were cultured with ILT-cell agonists in both a 7-day proliferation assay and ELISpot assay. At the end of 7-day culture, the fold change of each ILT-cell type following agonist stimulation was determined by flow cytometry. When healthy controls were compared to patients with prostate cancer, a significant reduction in 5-A-RU/MG-induced MAIT-cell expansion was observed (**Figure 2A**). Trends towards reduced NKT and Vγ9Vδ2 T-cell proliferation were also observed but did not reach statistical significance (**Figures 2B, C**). We observed no difference in the number of IFN-γ-producing cells detected by ELISpot assay following ILT-cell stimulation between healthy controls and patients (**Figures 2D–F**). Corticosteroids have previously been described to impair MAIT cell function (24), however a comparison of 5-A-RU/MG stimulated MAIT proliferation and IFN-γ ELISpot formation between patients receiving steroids and those not demonstrated no statistically significant differences (**Supplementary Figure 2**).

**Figure 3:**
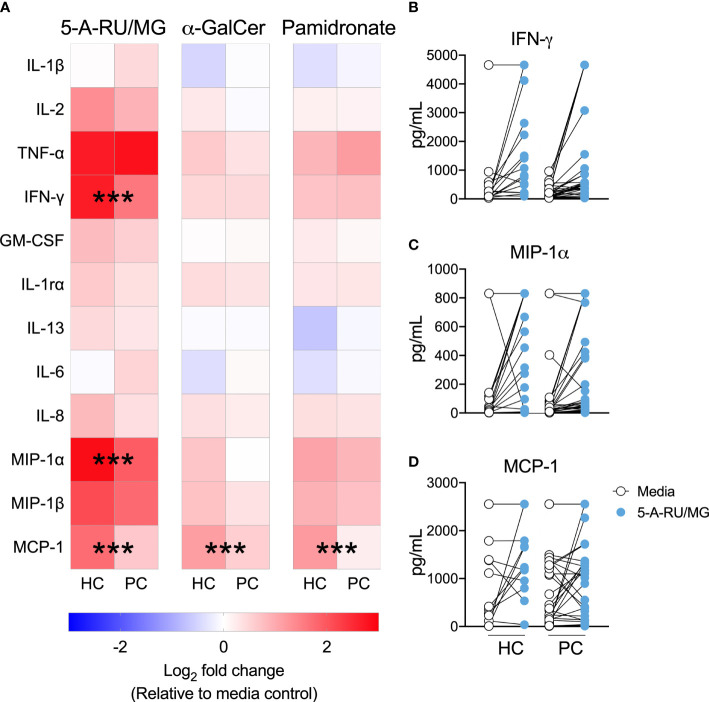
MAIT-cell agonist induced cytokines are impaired in men with prostate cancer. Culture supernatant was aspirated after 72 hours from the above proliferation assay and the Log_2_ fold change in cytokines above media control is displayed **(A)**. Multiple t-test. Individual plots quantifying IFN- **(B)**, MIP-1α **(C)** and MCP-1 **(D)** in 5-A-RU/MG stimulated and media control samples are also displayed. ***P < 0.001. N = 18 healthy controls (HC), 36 men with prostate cancer (PC).

ELISpot assays quantify the number of cytokine-producing cells, but not the amount of cytokine produced, or elicited, following MAIT-cell stimulation. Therefore, we used cytokine bead array to allow a comparison of cytokine quantity as opposed to cytokine spot forming unit frequency in the presence of various ILT-cell ligands. The production of IFN-γ, MIP-1α and MCP-1 in response to the MR-1 ligand 5-A-RU/MG was significantly impaired in patient PBMCs (**Figure 3**). In contrast, in response to the NKT ligand α-GalCer and indirect Vγ9Vδ2 ligand, pamidronate, only MCP-1 production was significantly impaired in patients compared to controls (**Figure 3**).

### Association Between Impaired MAIT-Cell Function and PD-1

**Figure 4:**
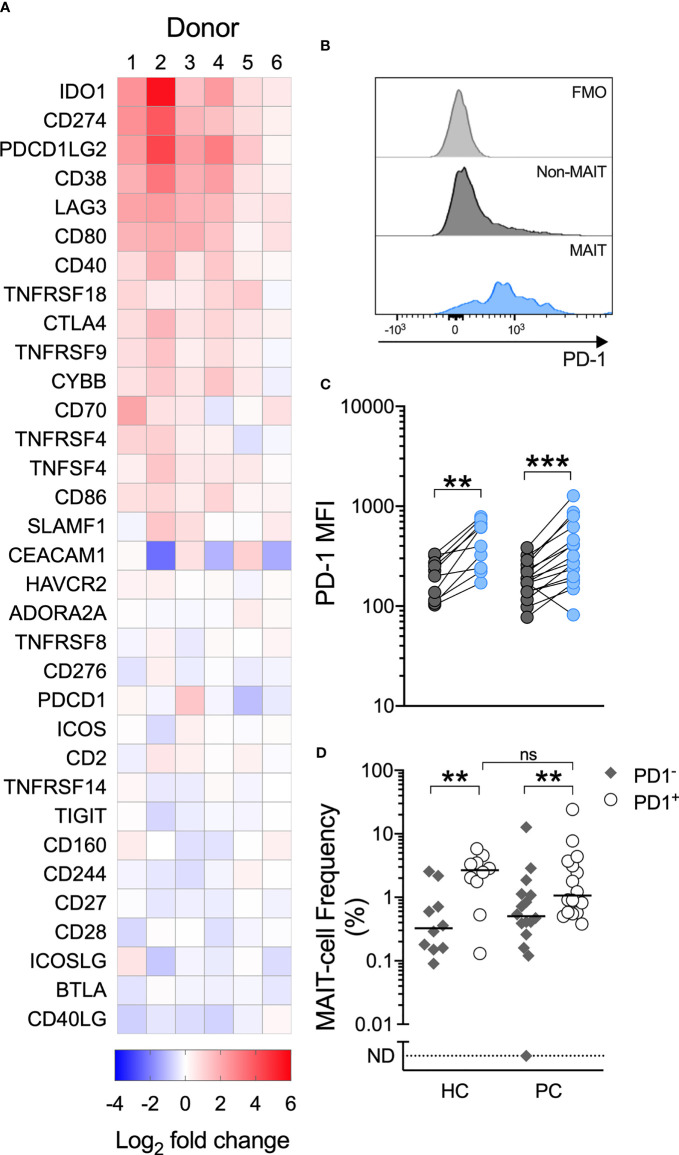
A MAIT-cell specific agonist upregulates PD-1 ligands, circulating MAIT-cells express high levels of PD-1 and MAIT-cells are over-represented among PD-1+ T-cells. Immune checkpoint genes significantly upregulated by PBMCs cultured with 5-A-RU/MG are shown in **(A)**. N = 6 healthy donors. Flow cytometric analysis was used to determine the surface expression of PD-1 on MAIT and Vα7.2TCR^-^CD161^+^/Vα7.2TCR^+^CD161^-^ T-cells in unstimulated PBMCs. Representative histograms **(B)** and surface PD-1 median fluorescence intensity (MFI) **(C)** are shown. The proportion of MAIT-cells within the PD-1^+^ and PD-1^-^ fractions of PBMCs from healthy controls and men with prostate cancer is displayed in **(D)**. N = 20 healthy controls (HC), 36 men with prostate cancer (PC). ns, no significance. **P < 0.01. ***P < 0.001. ND, non detectable. ns, no significance.

Having demonstrated impaired MAIT-cell proliferation and cytokine production in men with prostate cancer, we therefore characterized immune checkpoint molecule expression, which may influence MAIT-cell function. We used the NanoString PanCancer Immune panel to assess gene expression in PBMCs from 6 healthy donors stimulated with the MAIT-cell-specific agonist or vehicle. From this data we selected the expression of the 33 immune-associated inhibitory and stimulatory genes included in the panel for analysis. In response to 5-ARU/MG, we observed upregulation of the PD-1 ligands PD-L1 (*CD274*) and PD-L2 (*PDCD1LG2*), but not PD-1 (*PDCD1*) itself, across donors (**Figure 4A**). IFN-γ can induce expression of PD-1 ligands, while suppression of T-cell proliferation and IFN-γ production by PD-1 signaling has been well-described (25). We assessed the PD-1 expression on MAIT-cells in unstimulated PBMCs, and found higher expression compared to non-MAIT T-cells (**Figures 4B, C**). Accordingly, MAIT-cells were significantly over-represented among the PD-1^high^ fraction of total T-cells in both healthy controls and patients. There was no significant difference in the frequency of MAIT-cells within the PD-1^high^ fraction between patients and healthy controls (**Figure 4D**).

**Figure 5:**
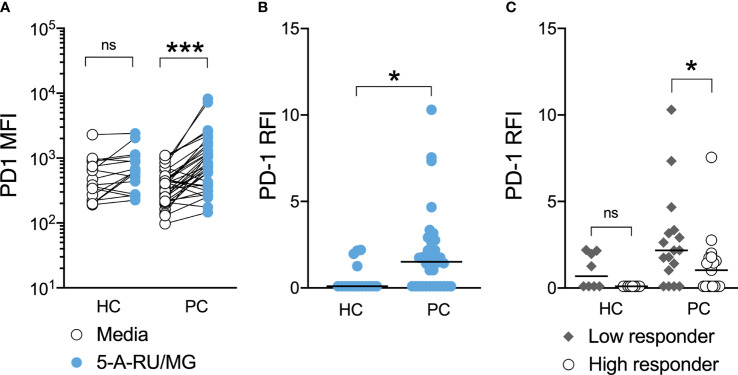
PD-1 upregulation is associated with impaired MAIT-cell proliferation in men with prostate cancer. At the end of 7-day culture in the above proliferation assay, the surface expression of PD-1 on stimulated and unstimulated MAIT-cells was compared **(A)** and the relative fluorescence intensity calculated **(B)** for healthy controls and men with prostate cancer. Participants were stratified into high and low responders according to the median MAIT-cell fold change for their respective groups and the PD-1 RFI compared **(C)**. Mann-Whitney test. N = 20 healthy controls, 36 men with prostate cancer. ns, no significance. *P < 0.05. ***P < 0.001.

Further, MAIT-cells from patients, but not healthy controls, significantly upregulated surface PD-1 following their stimulation with 5-A-RU/MG. When these groups were compared, we observed significantly greater upregulation of PD-1 on patient MAIT-cells (**Figures 5A, B**). To explore whether any relationship existed between proliferation and PD-1 upregulation, patients and healthy controls were divided into high and low responders on the basis of whether their proliferation in response to MAIT-cell agonist was above or below their group’s median proliferation, respectively. Patients, but not healthy controls, whose MAIT-cells underwent lesser proliferation in response to specific stimulation upregulated surface PD-1 to a greater extent (**Figure 5C**). Linear correlation of MAIT-cell proliferation versus MAIT-cell PD-1 Relative Fluorescence Intensity suggested a weak negative correlation between proliferation and PD-1 upregulation (**Supplementary Figure 3**; Spearman r = -0.3846, P-value = 0.0247).

### Anti-PD-1 Does Not Alter MAIT-Cell Function *Ex Vivo*

Having established that MAIT-cell dysfunction is associated with enhanced PD-1 expression in men with prostate cancer, we sought to assess the effect of PD-1 blockade on human MAIT-cells. On one hand, given the over-representation of MAIT-cells among PD-1^high^ T-cells (**Figure 4D**), pembrolizumab might deplete circulating MAIT-cells, and on the other hand it might enhance their function. As anti-PD-1 treatment is not approved for use in prostate cancer, we attempted to address this question in a cohort of 10 unresectable melanoma patients undergoing standard-of-care therapy with pembrolizumab. Men made up 80% of the patient group, 50% were receiving systemic steroids and no patients had received chemotherapy previously.

**Figure 6:**
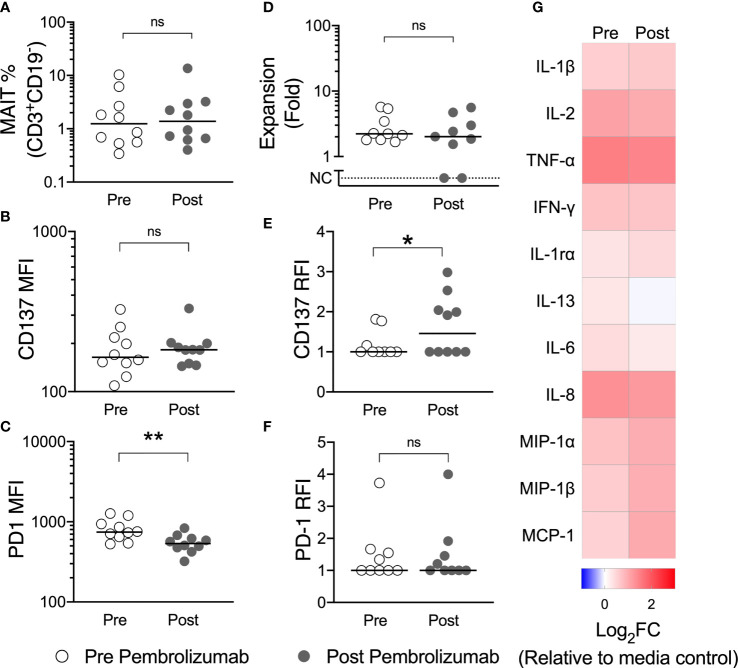
Pembrolizumab does not deplete MAIT-cells in vivo nor does it enhance MAIT-cell function ex vivo. The circulating frequency of MAIT-cells **(A)** and their surface expression of CD137 **(B)** and PD-1 **(C)** in unstimulated PBMCs was compared for patients with melanoma before and after their first dose of pembrolizumab. PBMCs were then cultured with 5-A-RU/MG for 7 days and the proliferation of MAIT-cells **(D)** and the upregulation of CD137 **(E)** and PD-1 **(F)** at the end of culture calculated. Mann-Whitney test. ns, no significance. *P < 0.05. **P < 0.01. No significant differences were observed for cytokines quantified in 72 hour culture supernatant for pre and post pembrolizumab samples **(G)**. N, 10.

Comparing pre-treatment with post-pembrolizumab samples, we observed a reduction in PD-1 expression, but no change in the frequency of circulating MAIT-cells or of the surface expression of the activation marker CD137 (4-1BB) on MAIT-cells in unstimulated PBMCs (**Figures 6A–C**). We believe the reduction in the detection of surface PD-1 likely reflects steric hindrance by the presence of pembrolizumab on cells, as has been previously demonstrated (26), but we did not evaluate this further. After stimulation with 5-A-RU/MG, we did not find a difference in *ex vivo* proliferation or cytokine production by circulating MAIT-cells following pembrolizumab therapy, although we did observe greater upregulation of CD137 on stimulated MAIT-cells after pembrolizumab treatment (**Figures 6D–G**).

### Anti-PD-1 and MAIT Cell Activation Controls Prostate Cancer Growth *In Vitro*

**Figure 7:**
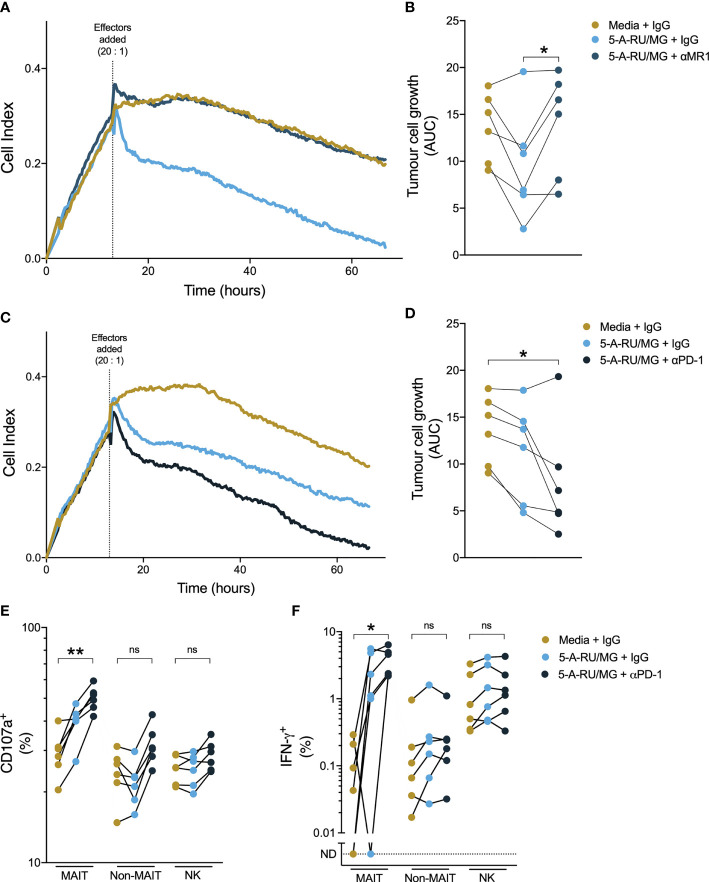
Anti-PD-1 enhances healthy donor MAIT-cell suppression of prostate tumour growth in vitro. PC3 cells were grown in a real-time impendence based cell killing assay for 12 hours in media control or 5-A-RU/MG prior to the addition of healthy donor PBMCs. The effects of anti-MR1 **(A, B)** and anti-PD-1 **(C, D)** on tumor cell growth were calculated as the area under the curve. PC3 cells were primed with 5-A-RU/MG or media control and co-cultured with healthy donor PBMCs overnight and the CD107a degranulation **(E)** and IFN-γ expression **(F)** were calculated by flow cytometry. Friedman test with Dunn’s multiple comparison test. ns = no significance. *P < 0.05. **P < 0.01. N = 6 healthy donors.

While therapeutic PD-1 blockade had little impact on the number or function of circulating MAIT-cells, we wished to establish whether anti-PD-1 could affect MAIT-cell function in the setting of tumour cells expressing PD ligands. To do so, we employed an impendence-based assay to assess cytotoxicity against the prostate cancer cell line PC3, which expresses PD-L1 (27). We found that healthy donor PBMCs suppressed growth of PC3 in response to 5-A-RU/MG, and that this suppression was MR-1-dependent (**Figures 7A, B**). Pre-treatment of healthy donor PBMCs with anti-PD-1 further enhanced 5-A-RU/MG suppression of PC3 growth (**Figures 7C, D**). Finally, when we treated PC3 cells with 5-A-RU/MG, then co-cultured with pembrolizumab or isotype control-treated healthy donor PBMCs, we found that a greater proportion of MAIT-cells, but not non-MAIT cells or NK-cells, expressed the cytotoxicity-associated molecules CD107a and IFN-γ (**Figures 7E, F**).

## Discussion

Using samples from a cohort of men with prostate cancer and age- and sex-matched controls, we report that circulating MAIT-cells are numerically preserved in prostate cancer but exhibit impaired cytokine production and proliferative capacity in response to the MR-1 ligand 5-A-RU. We find that MAIT-cells express high levels of PD-1 at rest, that MAIT-cell PD-1 expression is enhanced by 5-A-RU, particularly in patients, and that 5-A-RU leads to upregulation of PD-L1 and PD-L2 RNA within mononuclear cells. We find that despite high MAIT-cell PD-1 expression, clinical exposure to the humanized anti-PD1 antibody pembrolizumab does not deplete circulating MAIT-cells. Finally, we report that 5-A-RU elicits MR-1-dependent lysis of a prostate cancer cell line by human PBMCs *in vitro*, and that this effect is enhanced by pembrolizumab, in association with increased MAIT-cell expression of the cytotoxicity-associated molecules CD107a and IFN-γ.

To our knowledge, this study is the first to describe MAIT-cell numbers and function in prostate cancer. As well as describing MAIT-cell dysfunction in patients, we associate this with enhanced PD-1 upregulation upon specific stimulation, and demonstrate that PD-1 blockade can lead to enhanced MAIT-cell cytotoxicity against a prostate cancer cell line in response to a MR-1 ligand. Moreover, in a separate cohort of patients with melanoma, we report that treatment with pembrolizumab does not deplete, nor impede the function of, MAIT cells.

Our patient cohort was of similar size to other clinical cohorts examining MAIT-cell number and function (28, 29), and we were careful to age- and sex-match the patient and control groups. However, the patients were not uniformly treated, and our sample size is insufficient to explore associations between disease stage or specific treatments on MAIT-cell number and function. The cross-sectional study design does not allow us to determine whether MAIT-cell dysfunction is involved in prostate cancer onset or progression or a consequence of prostate cancer therapies – longitudinal studies could provide insight into this.

We identified MAIT-cells as cells expressing the Vα7.2 TCR chain, CD161, and CD3. While this is considered one of the most reliable surrogate staining methods for human MAIT-cells, compared to MR1 tetramer staining, we may have overestimated CD4^+^ MAIT-cells, and our staining method would not have identified either MR1-restricted T-cells that do not use the Vα7.2 chain, or the small population of CD161^-^ MAIT-cells (30, 31). The functional assays we report, such as cytokine production and cytotoxicity in response to 5-A-RU, are not affected by this limitation. We characterized only circulating MAIT-cells. It is plausible that MAIT-cells within the prostate tumor microenvironment differ in terms of number, cytokine profile or proliferative capacity. For example, Ling et al. reported a reduction in circulating MAIT-cells, but an increase in tumor-infiltrating MAIT-cells, in colorectal cancer (28).

Although MAIT-cells have not been previously studied in prostate cancer to our knowledge, they have been studied in other malignancies. Circulating MAIT-cell numbers were reduced in patients with esophageal cancer (29), whereas there is conflicting evidence in colorectal cancer (28, 32, 33). Reduced cytokine production, including of IFN-γ, by circulating and intra-tumoral MAIT-cells has been reported in patients with both colorectal (28, 32) and hepatocellular carcinoma (34), consistent with our own observation. Innate-like T-cells other than MAIT-cells have been studied in prostate cancer: Tahir et al. described a functional NKT deficit and an inability to detect circulating NKTs in a small cohort of men with prostate cancer (22). In our larger patient cohort, we did not see a difference in circulating NKT numbers, although we observed a non-significant trend towards reduced NKT expansion. Normal circulating levels of Vδ2 T-cells have previously been reported in prostate cancer (23). consistent with our own finding in the more specific Vγ9Vδ2 T-cell subset.

Clinical trials of immunotherapy with PD-1 blockade have had limited success in prostate cancer to date (7–9). A recent report found that higher MAIT-cell numbers and greater MAIT-cell expression of cytotoxicity-associated molecules predicted clinical response among patients receiving anti-PD-1 therapy for melanoma (35). In conjunction with our own finding that MAIT-cells are highly over-represented among PD-1^high^ T-cells, this raises the possibility that MAIT-cells are directly involved in clinical responses to therapeutic PD-1 blockade.

In keeping with their capacity to recognize microbial metabolites, MAIT-cells are present at high frequencies in barrier tissues (36), including in the urinary tract (16, 20). Although T-cell infiltration into prostate cancer is characteristically limited (37), the MAIT-cell-associated Vα7.2-Jα33 TCR transcript has been identified in human prostate tissue (15). Moreover, in an analysis of public datasets, gene expression signatures associated with MAIT-cells were identified in prostate cancer, correlated with MR-1 expression, and were associated with a favorable prognosis (21).

The most frequent human urinary tract pathogens, *E. coli* and *Klebsiella* species (38), produce riboflavin metabolites that result in potent MAIT-cell activation, and dysfunction of MAIT-cells has been described among patients with recurrent urinary tract infections (20). Intriguingly, in a mouse model of prostate cancer, the combination of a uropathic strain of *E. coli* (administered intraurethrally) with systemic anti-PD-1 therapy resulted in improved T-cell infiltration, prostate cancer regression and survival (39). In conjunction with our own finding that a potent MR-1 ligand and pembrolizumab can co-operate to enhance MR-1-dependent cytotoxicity against a prostate cancer cell line, this raises the possibility of rationally combining PD-1 blockade with prostate-directed (intraurethral or transrectal) administration of MR-1 ligands. By recruiting, activating and de-repressing MAIT-cells, this combination might turn the cold prostate cancer microenvironment hot (37). *In vivo* assessment of this strategy would be necessary: although most mouse strains have few MAIT-cells compared to humans, xenograft models, or models based on the MAIT cell-rich CAST/EiJ mouse strain could be employed (16).

In conclusion, we identify normal numbers, but impaired function, of circulating MAIT-cells in patients with prostate cancer. We find that this MAIT-cell dysfunction is associated with enhanced PD-1 expression upon stimulation, and that the combination of a potent MR-1 ligand with PD-1 blockade enhances MR-1-dependent cytotoxicity against a prostate cancer cell line. Our findings support investigation of PD-1 blockade in combination with MAIT-cell stimulation for the treatment of prostate and other cancers.

## Data Availability Statement

The RNA Nanostring data discussed in this publication have been deposited in NCBI's Gene Expression Omnibus (40) and are accessible through GEO Series accession number GSE184313 (https://www.ncbi.nlm.nih.gov/geo/query/acc.cgi?acc=GSE184313).

## Ethics Statement

The studies involving human participants were reviewed and approved by the Victoria University of Wellington Human Ethics Committee (Ref: 20957) and the University of Otago Ethics Committee (Ref: H15/114). The patients/participants provided their written informed consent to participate in this study.

## Author Contributions

E-MJ, SC, and RW designed experiments, applied for ethics, and collected and analysed the data. AA-H and ND assisted with assay troubleshooting, data acquisition and analysis. BL and JN provided clinical guidance for and facilitated the recruitment of patients with cancer. GP oversaw the design, synthesis and stabilisation of 5-A-RU and α-GalCer. BD, IH, and RW provided oversight for ethics, experimental design and statistical analysis. E-MJ and RW wrote the manuscript and prepared the figures, which were reviewed by all authors prior to submission. All authors contributed to the article and approved the submitted version.

## Funding

Funding for the above project was provided by the Prostate Cancer Foundation of New Zealand, the Health Research Council of New Zealand (14/502), the Wellington Division of the Cancer Society of New Zealand, and the Thompson Family Foundation.

## Conflict of Interest

The authors declare that the research was conducted in the absence of any commercial or financial relationships that could be construed as a potential conflict of interest.

## Publisher’s Note

All claims expressed in this article are solely those of the authors and do not necessarily represent those of their affiliated organizations, or those of the publisher, the editors and the reviewers. Any product that may be evaluated in this article, or claim that may be made by its manufacturer, is not guaranteed or endorsed by the publisher.
