# Supplementary material for: Mucosal-Associated Invariant T (MAIT) Cell Dysfunction and PD-1 Expression in Prostate Cancer: Implications for Immunotherapy
Source: Front Immunol. 2021 Oct 19;12:748741. doi: 10.3389/fimmu.2021.748741 (PMC8560687; doi:10.3389/fimmu.2021.748741)
Supplement: Supplementary file 1 [file DataSheet_1.docx]

Supplementary Material

**Supplementary Table 1:** Comparison of male healthy controls and men with prostate cancer

| Characteristic | Proportion of Controls  (n = 20) | Proportion of Patients  (n = 36) | P-value |
| --- | --- | --- | --- |
| Median Age  (IQR) | **71**  (67.25 — 76) | **70.5**  (64.25 —75) | **0.33^†^** |
| Systemic corticosteroid use  (%) | **0**  (0) | **9**  (25) | ***0.0195^‡^** |
| Aminobisphosphonate use  (%) | **1**  (5) | **1**  (2.8) | **>0.99^‡^** |
| Statin use  (%) | **8**  (40) | **13**  (36.1) | **0.78^‡^** |
| Other Immunosuppressant  (%) | **0**  (0) | **0**  (0) | **>0.99^‡^** |
| IQR = interquartile range. ^†^P-value determined by Unpaired t-test. ^‡^P-value determined by Fisher’s exact test. *Denotes p-value <0.05. | | | |

**Supplementary Figure 1:** *Flow cytometric gating strategy for the identification of NKT, MAIT and V𝛾9V𝛿2 T-cells from PBMCs.* Fully stained samples are depicted in the second row, with the respective fluorescence minus one (FMO) control for each ILT-cell TCR beneath them.

Supplementary Figure 2: *No difference in patient MAIT-cell function with systemic steroid use. 5-A-RU/MG stimulated MAIT expansion at the end of 7 days and IFN-𝛾 ELIspot formation after 48 hours is displayed. Mann-Whitney test. N = 27 patients not receiving steroids, 9 patients receiving steroids. ns = no significance.*

Supplementary Figure 3: *Correlation of MAIT-cell proliferation to PD-1 upregulation. MAIT‑cell proliferation was plotted against MAIT-cell PD-1 RFI as determined at the end of 7‑day culture following stimulation with 5-A-RU/MG. Line indicates simple linear regression. Spearman correlation.*
